# Mechanism design for a fair and equitable approach to global vaccine distribution: The case of COVID-19

**DOI:** 10.1371/journal.pgph.0001711

**Published:** 2023-12-28

**Authors:** Khaled Abedrabboh, Lolwa Al-Majid, Zaid Al-Fagih, Luluwah Al-Fagih

**Affiliations:** 1 Division of Sustainable Development, College of Science and Engineering, Qatar Foundation, Hamad Bin Khalifa University, Doha, Qatar; 2 Division of Engineering Management and Decision Sciences, College of Science and Engineering, Qatar Foundation, Hamad Bin Khalifa University, Doha, Qatar; 3 Rhazes AI, London, United Kingdom; 4 School of Computer Science and Mathematics, Kingston University, London, United Kingdom; Johns Hopkins University Bloomberg School of Public Health, COLOMBIA

## Abstract

Vaccines are one of the most effective tools humanity has in the fight against pandemics. One of the major challenges of vaccine distribution is achieving fair and equitable allocation across the countries of the world, regardless of their economic wealth. The self-interested behaviour of high-income countries and the underutilisation of vaccines allocated to underprepared countries are some of the failures reported during COVID-19 vaccine roll-out. These shortcomings have motivated the need for a central market mechanism that takes into account the countries’ vulnerability to COVID-19 and their readiness to distribute and administer their allocated vaccines. In this paper, we leverage game theory to study the problem of equitable global vaccine distribution and propose a fair market mechanism that aligns self-interested behaviour with optimal global objectives. First, we model the interaction between a central vaccine provider (e.g. COVAX) and a country reporting its demand as a two-player game, and discuss the Nash and mixed Nash equilibria of that game. Then, we propose a repeated auction mechanism with an artificial payment system for allocating vaccines among participating countries, where each auction round is based on a *Vickrey-Clarke-Groves* (VCG) mechanism. The proposed allocation mechanism aims at minimising deaths and incentivises the self-interested countries to report their demand truthfully. Compared with real-world COVAX allocation decisions, our results show that the proposed auction mechanism achieves more efficient outcomes that maximise the number of averted deaths. Pragmatic considerations are investigated and policy recommendations are discussed.

## 1. Introduction

Epidemics can cause severe damages on a global scale. Numbers of deaths and cases with long term health consequences rise exponentially at the onset of an outbreak [1]. Humanity has, primarily, two effective tools to limit the spread of a large-scale pandemic. The first is to introduce non-pharmaceutical interventions (NPI) such as lockdowns and physical distancing. These have helped in suppressing the transmission of the recent COVID-19 pandemic [2]. However, they have also had crippling effects on economies, societies, and inidividuals’ mental health [3]. The other tool in the fight against pandemics is the development and broad administration of vaccines. Their benefits are not limited to the individuals that receive them. Indeed, by significantly reducing virulence, transmission, hospitalisations and deaths, they hold the potential to allow for the lifting of NPI’s, thus providing economic, psychological and social benefits [4, 5].

One of the major challenges that are faced with regards to vaccine distribution is achieving fair and equitable allocation across the countries of the world, regardless of their economic power [6]. There are both ethical and public health arguments for equitable vaccine distribution. Not only does it help protect the most vulnerable, but it also limits the disproportionate harm that economically-deprived populations face during a pandemic [7]. There is also an argument [8] supporting equitable vaccine distribution based on the self-interest of wealthy countries. In a globally interconnected world, infectious diseases rarely respect political borders. It is therefore crucial to ensure vaccination is as widespread as possible to reduce transmission on a global scale. Moreover, the higher the circulating levels of a viral pandemic, the higher the probability of the emergence of a vaccine-evading variant [9]. Thus, providing low-income countries with equitable access to vaccines is a necessary global objective. COVAX is a global initiative [10] that aims to achieve that objective for COVID-19 vaccines. Launched in April 2020, its initial fair allocation framework had two phases; allocating its secured vaccines proportionally to vaccinate 20% of all participating countries’ populations, and then allocating to countries according to their vulnerability and need [11]. Whilst proportional allocation is considered as a fair approach to vaccine distribution, it goes against the fundamental function of vaccination: minimising deaths.

Using a metric that is representative of the threat of COVID-19 to a country has greater benefits than using its population count in making its allocation decision. It can better realise global equity and distributive justice [12]. Another metric that should be inherent in the allocation mechanism is the countries’ preparedness and readiness to administer vaccines within its population. Given the scarcity of vaccine supply, it is necessary to prevent the delivery of vaccines that would be wasted in underprepared locations [13]. Scarcity of COVID-19 vaccines has also led the countries with political and financial influence to seek bilateral supply deals with the vaccine manufacturers. This was detrimental to the point that by June 2021, 75% of all produced vaccines had been administered in only 10 countries [14]. Thus, manifesting the need for a central market mechanism that takes into account the countries’ vulnerability to COVID-19 and their readiness to distribute and administer their allocated vaccines.

Game theory is a tool that can be used to analyse the interactions of the various actors in the vaccine distribution problem. In this context, each participating country can be modelled as a self-interested or ‘selfish’ rational agent that wishes to maximise its benefit from receiving vaccination doses. On the other hand, the COVAX facility can be modelled as a principal agent that has the power to influence the game and design the market rules so that the desired outcome of achieving equitable distribution of vaccines can be realised.

Game theory has been used to study optimal resource planning within healthcare facilities. Wu [15] proposed a game-theoretic approach to prioritisation in an emergency department (ED). In their framework, emergency events are classified through a non-cooperative game between patients reporting their urgency levels to the ED administrator. The competition of these event categories on ED resources is then likened to a cooperative game where each category is considered as a coalition of emergency events. The ED administrator then uses the Shapley value of each event to allocate and schedule the ED resources to these events. Adida *et al*. [16] investigated the problem of resource stockpiling in hospitals from a game-theoretic perspective. The authors modeled the strategic behaviour of self-interested hospitals, who wish to minimise their stockpiling cost, as a non-cooperative game. A shortage penalty is introduced as an incentive for hospitals to increase their stockpiles. Lofgren *et al*. [17] extended this approach by accounting for network capacity constraints. Using a real-world hospital network from North Carolina, USA, the authors found that shortages in supply can be significant if the applied shortage penalty is low. More recently, Abedrabboh *et al*. [18] developed a dynamic game for healthcare facilities to stockpile and schedule their personal protective equipment (PPE) orders so that severe shortages can be avoided during an epidemic. In their proposed centralised-decentralised approach, a demand-dependent cost function for PPE is used as an incentive for healthcare facilities to optimise their PPE storage utilisation, thus limiting demand spikes. The competition between countries to procure medical supplies during COVID-19 was modelled as a stochastic game in [19]. In this proposed scheme, competing countries aim to minimise their disutility, which comprises of procurement costs, transportation costs, and the uncertain consequences of supply shortages. The authors argue that the ability to increase domestic supply when disasters occur can have significant effects on the countries’ success in managing such disasters.

Others have proposed game-theoretic approaches to analyse the effect of vaccine distribution on epidemic control. [20, 21] have compared decentralised ‘Nash’ vaccination to centralised ‘utilitarian’ vaccination and studied their impact on epidemics. In [20], the effects of these vaccination strategies and the effect of vaccine cost on infections in an epidemic were used to suggest policies for vaccination subsidies and awareness. On the other hand, the authors in [21] developed a two-player game to model the interactions between two countries that compete for medical resources in an epidemic. The authors found that infections are minimised when the two countries share their resources and a central planner allocates those resources according to the epidemiological parameters of each country.

More relevantly, [22, 23] suggested market designs for the fair distribution of COVID-19 vaccines. Akbarpour *et al*. [22] have taken a mechanism design approach to investigate whether to adopt prices or priorities when allocating vaccines within a country. The authors distinguished between the use of observable characteristics (e.g. age and profession) and unobservable (private) characteristics (e.g. social and economic benefits) of an agent to propose optimal vaccine allocation mechanisms. Although this study suggests policies on whether to deregulate the vaccine market within a country, it cannot be translated to a global context where the distribution of wealth is highly asymmetric. Pancs [23] has developed an auction mechanism to schedule vaccination among a country’s population. The proposed auction asks agents to report their valuations of getting vaccinated. It also allows them to bid their valuations of getting others vaccinated. All agents are then queued for vaccination according to the aggregate valuation they get from all bids.

Although this proposed mechanism provides a method to deal with the externalities of vaccination, it is vulnerable to manipulation by immoral and spiteful bidding behaviour. This is because agents are allowed to submit negative valuations to represent their disutility of getting themselves and others vaccinated (e.g. vaccine skeptics and people who are allergic to vaccines). Additionally, the practicality of such a mechanism is questionable as the number of bids and the dimension of each bid can both exceed the population size.

In this paper, we leverage game theory to study the problem of equitable global vaccine distribution and propose a fair market mechanism that aligns selfish objectives with optimal global objectives. First, we model the interaction between a central vaccine provider (e.g. COVAX facility) and a country reporting its demand as a two-player game, and discuss the Nash and mixed Nash equilibria of that game. Then, we take inspiration from [24] and propose a repeated auction mechanism without payments for allocating vaccines among participating countries. Each iteration of this repeated auction is based on *Vickrey-Clarke-Groves* (VCG) mechanism with artificial payment. The proposed allocation mechanism aims at minimising deaths and incentivises the self-interested countries to report their demand truthfully. Using real world COVID-19 data, we compare our allocation results with the allocation decisions made by COVAX in Round 7 (announced on 17 September 2021) [25] and investigate pragmatic considerations. Finally, we draw conclusions and discuss future research directions. Our contributions can be summarised as follows:

1. An investigation of the key criteria that should be evaluated for the equitable distribution of vaccines in a global context; and proposing a novel utility function that represents the benefits of receiving vaccine doses.
2. The design of a two-player game modeling the interaction between a central vaccine provider, such as the COVAX facility, and a country reporting its demand for vaccines based on its distribution readiness; and an analysis of the pure and mixed Nash equilibria, motivating our proposed VCG methodology.
3. A novel repeated auction mechanism for allocating vaccines with the objectives of minimising deaths and preventing strategic malicious behaviour by employing an artificial payment system with budget allocation.
4. Suggestions and insights into how the proposed central allocation mechanism can be adopted in practice, thus informing on how policies can be made to implement and regulate the proposed vaccine market in the current vaccination drive led by COVAX.

The remainder of this paper is organised as follows. In Section 2 we discuss the key factors that should be used to evaluate fairness of vaccine distribution, and provide a utility function formulation that reflects these factors. We formulate the two-player game in Section 3 and analyse its equilibria. Section 4 develops the proposed allocation mechanism and discuss its properties. Our results are presented in Section 5 and compared with the reference COVAX allocations of Round 7 made in September 2021. Practical considerations and policy recommendations are provided in Section 6. Section 7 concludes the paper and indicates possible future research directions.

## 2. Problem statement

In this section, aspects relating to the vaccine distribution problem are highlighted and the benefits of vaccination are discussed. Then, we formulate a country’s utility of receiving vaccines as a function of its recent death count and its readiness to administer vaccines locally. Finally, some of the fair division methods that are proposed in the literature are analysed in the context of the vaccine distribution problem.

### 2.1 Efficient allocation

Vaccines help reduce the risk of deadly diseases. In the past, they have helped in the elimination of polio and the eradication of smallpox [26]. They hold the potential to protect humanity against morbidity and mortality from current and future epidemics [27]. Nonetheless, the distribution and allocation of vaccines to where they are needed the most in the world is a challenge. This work is concerned with finding a global market mechanism that allocates vaccines fairly and that maximises the benefits of vaccine distribution. In order to evaluate the fairness criteria that should be used to guarantee equitable distribution of vaccines, we need to analyse the benefits countries get when receiving these vaccines. First and foremost, the main benefit of getting vaccinated is to reduce the possibility of death or hospitalisation from an illness related to an epidemic. Another major benefit is that vaccinated individuals are less likely to be infected, which in turn reduces the chances of disease transmission to other individuals. Thus, the more people are vaccinated within a country the more the reproduction number decreases. Vaccines also have economic, security, and social benefits. A country with a relatively low reproduction number will relax NPI restrictions, thus allowing the economic recovery of some of the industries that are most impacted by epidemics (e.g. air travel and hospitality [28]). Vaccines’ economic benefits also include reduced healthcare spending due to less hospitalisations and less strain on healthcare supply chains. Recent epidemics have also highlighted the impact of vaccination on energy and food security [29–32]. Social benefits will normally result from the relaxed NPI restrictions. Due to the complexity of the benefits and externalities that countries receive from procuring vaccines, we restrict our model to the most direct result of vaccinating a population: reducing deaths. Nonetheless, the expected number of deaths averted in a country is not only influenced by the number of received vaccination doses, but it also depends on the ability of that country to distribute and administer these doses.

### 2.2 Utility function formulation

We assume that the benefit a country gets from receiving vaccination doses can be represented by a utility function *u*. We argue that the main objective a country wishes to achieve from acquiring vaccines is to reduce the number of deaths. This can be modelled as a function of three variables:

- Scalar state variable *D* denoting the recent death rate, i.e. the number of the recently reported deaths that are caused by the epidemic in question, normalized per population count.
- Scalar state variable *R* denoting the number of vaccination doses that the country is ready to distribute and administer in a given time horizon. We will use *readiness* to refer to this variable hereafter.
- Scalar decision variable *q* denoting the number of received vaccination doses.

Thus, utility is a function of two characteristic variables that are known to the country and can be coupled together in type vector *θ* where *θ* = *θ* (*R*, *D*). It is also a function of the number of received vaccination doses *q*, which is a variable that is determined by the mechanism. The following assumptions describe the relationship between the utility function *u*(*q*, *θ*) and these variables:

1. No utility is yielded when no vaccination doses are received, i.e.:
  $$\begin{array}{c} u(0,\theta )=0. \end{array}$$ (1)
2. The utility function *u*(*q*, *θ*) is non-decreasing in *q*, i.e. an increase in the amount of received vaccines will not lower utility:
  $$\begin{array}{c} \frac{\partial u(q,\theta )}{\partial q}\geq 0. \end{array}$$ (2)
3. The marginal utility is non-increasing in *q*. This means that the utility gained from a single dose will be lower as the number of received doses increases. This stems from the law of diminishing marginal utility, which is discussed at length in [33].
  $$\begin{array}{c} \frac{\partial^{2}u\left(q,\theta \right)}{\partial q^{2}}\leq 0. \end{array}$$ (3)
4. The higher the death rate in a country, the higher its utility of getting vaccines, i.e.:
  $$\begin{array}{c} \frac{\partial u(q,\theta )}{\partial D}\geq 0. \end{array}$$ (4)

These assumptions can be satisfied by modelling the utility function as a concave function in *q*. Additionally, a country with a high death rate will gain more utility from receiving vaccines compared to a country with a lower death rate. Furthermore, when a country gets more vaccines than its readiness, these surplus vaccines will have little to no value in terms of saving lives. However, given that a country may mispredict its readiness, or that it could receive aid in vaccine administration, surplus vaccines may generate utility. Nonetheless, the marginal utility of any surplus vaccines is expected to be lower than that of vaccines that will surely be used, i.e. when the received vaccination doses are less than or equal to readiness. These characteristics can be represented by the following piece-wise linear utility function:

$$\begin{array}{c} u\left(q,\theta \right)=\{\begin{array}{cc} Dq & \;q\leq R\\ (\frac{\alpha }{\beta }\left(\frac{q}{R}-1\right)+1)DR & \;R\leq q\leq (1+\beta )R\\ (1+\alpha )DR & \;q\geq (1+\beta )R, \end{array} \end{array}$$ (5)

where *α* and *β* are predetermined parameters that represent the increase in utility *αDR* from any surplus vaccines *β*. Piece-wise linear utility function formulations were used in the context of energy (see [34] and the references therein), computing [35, 36], and behavioural economics [37, 38]. To the best of our knowledge, this is the first study that proposes a piece-wise linear function formulation to represent the utility countries get when receiving vaccine allocations.

### 2.3 Fairness criteria

This work aims to design a market mechanism that allocates vaccines among countries fairly. The literature on fair division of limited resources investigates four allocation mechanisms [39]. Assuming that there are *N* number of agents (i.e. countries) where N={1,…,N} is the set of all agents, these allocation mechanisms are formulated as follows:

- **Proportional division**: each agent gets at least the average utility from their own perspective,
  $$\begin{array}{c} u_{i}\left(q_{i}\right)\geq u_{i}\left(\sum_{i}^{N}q_{i}\right)/N\;\;\;\forall i\in N. \end{array}$$ (6)
- **Envy-free division**: each agent gets a share that is at least as valuable as any other agent’s share,
  $$\begin{array}{c} u_{i}\left(q_{i}\right)\geq u_{i}\left(q_{j}\right)\;\;\;\forall i,j\in N. \end{array}$$ (7)
- **Egalitarian (equitable) division**: all agents get the same utility,
  $$\begin{array}{c} u_{i}\left(q_{i}\right)=u_{j}\left(q_{j}\right)\;\;\;\forall i,j\in N. \end{array}$$ (8)
- **Utilitarian division**: agents get allocations that maximise the social welfare (i.e. sum of utilities [40])
  $$\begin{array}{c} q^{*}\in \underset{q}{\text{arg max}}\sum_{i\in N}u_{i}\left(q_{i},\theta_{i}\right). \end{array}$$ (9)

To simplify the problem, we assume that all vaccination doses from all manufacturers have the same efficacy against death in expectation, i.e. produce equal expected value to the countries that receive them. Thus implying that vaccine is a homogeneous good. However, this is not necessarily a limitation as it directly applies to infectious diseases with vaccines that require a single shot or a single type of vaccine. In the context of allocating a homogeneous good, and given that our objective is to minimise deaths globally, we need only concern ourselves with optimising the social welfare (i.e. utilitarian division). Proportional and envy-free division are mostly applicable to heterogeneous goods [39], while egalitarian division often leads to inefficient outcomes [41]. Nonetheless, calculating the optimal allocations from Eq 9 requires knowledge of the individual characteristics of all agents $\theta_{i}\forall i\in N$. One could argue that the recent death rate *D* is an observable characteristic that can be considered as public information. However, the other component of *θ*, readiness *R*, can only be estimated locally within a country and thus can only be considered as private information. Therefore, in order to solve the optimal social welfare problem, we need to ask the participating countries to self-report their respective readiness. This can lead to gaming opportunities where selfish countries seek to maximise their utility by misreporting their readiness. Section 3 discusses this limitation in the context of a two-player game.

## 3. A two-player game formulation

This section presents a brief game-theoretic formulation of the interaction between a central vaccine allocation decision maker such as the COVAX facility and a country reporting its vaccine demand based on its readiness to distribute and administer vaccines locally. We take inspiration from a well-known class of two-player security games between an attacker and a defender (see for example [42] and references therein) in which an attacker may, or may not, attack a system e.g. by injecting false information into it, and the defender must decide whether to defend by monitoring the system (and bear the associated costs), or not. The VIRAT/VRAF 2.0 tool [43], developed by COVAX and the World Bank to measure readiness of vaccine deployment, is heavily reliant on each country self-reporting their readiness data and thus exposes the COVAX facility to the risk of self-misreporting. We seek to combat this by designing a two-player nonzero-sum game between the COVAX facility F and an agent country A. In the proposed game, the agent country can be either truthful, or deceptive, in reporting their demand for a certain quantity of vaccines (i.e. their readiness to distribute and administer them); this is denoted by the strategy space $S_{A}=\left\{Truthful,Deceptive\right\}$. The COVAX facility will in turn either trust or verify this information: $S_{F}=\left\{Trust,Verify\right\}$. If the country is truthful, and the COVAX facility decides to trust, the country will benefit from meeting its demand, *b*. If the COVAX facility chooses to verify, the country’s demand *b* will be met, but the COVAX facility will bear the additional cost of verification *c*. On the other hand, if the country chooses to be deceptive (e.g. by over-reporting their true readiness in order to receive a higher portion of vaccine dose allocations) and the COVAX facility chooses to trust, the country will receive the amount ordered *d*, but this will come at a cost, *e*, to the COVAX facility who could have allocated the extra vaccines to another country that is ready to distribute them. If in this scenario the COVAX facility decides to verify, the country will receive an allocated quantity *b* (i.e. its true demand) and pay a penalty amount *a* for misreporting. The COVAX facility will incur the cost of verification *c*. We assume that the penalty imposed by the COVAX facility always exceeds the cost of verifying, i.e. *a* > *c*; and that the benefit from receiving a higher allocation of vaccine doses exceeds that of receiving a lower number of doses, i.e. *d* > *b*. The game is illustrated in Table 1 whereas the definitions of positive parameters *a*, *b*, *c*, *d*, and *e*, all in monetary units, are listed in Table 2.

**Table 1:** COVAX game in normal form.

		COVAX facility F	
		*Trust*	*Verify*
Country A	*Truthful*	*b*, 0	*b*, −*c*
	*Deceptive*	*d*, −*e*	*b* − *a*, −*c* + *a*

**Table 2:** Parameters in the vaccine allocation game.

Parameter	Definition
*a*	Penalty
*b*	Benefit of meeting true demand
*c*	Verification cost
*d*	Benefit of meeting untrue demand
*e*	Value of wasted vaccines

### 3.1 Nash equilibrium discussion

It can be shown (see e.g. [44]) that there are no pure Nash Equilibrium strategies unless one or more of our assumptions are broken. This means that the agent country or the COVAX facility may always have the incentive to change their strategy in return for a higher payoff. This is not inline with our aim to arrive at a strategy that ensures that the agent country always reports its demand truthfully, and reduce the verification costs for the COVAX facility. If, however, the two players choose to play randomly, i.e. the COVAX facility chooses to *Trust* with probability *p* and the agent country chooses to be *Truthful* with probability *q*, then we can compute the probabilities p* and q*, that form a mixed Nash equilibrium strategy $\left(S_{F}^{*},S_{A}^{*}\right)$. This computation gives p* = *a*/(*d* − *b* + *a*) and q* = (*e* − *c* + *a*)/(*a* + *e*), where the conditions in Eqs 10 and 11 must be imposed to ensure that p* and q* can be used as a probability:

$$\begin{array}{c} 0\leq \frac{a}{d-b+a}\leq 1, \end{array}$$ (10)

$$\begin{array}{c} 0\leq \frac{e-c+a}{a+e}\leq 1. \end{array}$$ (11)

Given our game assumptions, we can see that conditions (10) and (11) always hold, i.e. there exists a mixed Nash equilibrium strategy $\left(S_{F}^{*},S_{A}^{*}\right)$ such that the COVAX facility trusts with probability p* and the agent country is truthful with probability q*. This exposes the COVAX facility to the risk of the agent country being *Deceptive* with likelihood 1 − q* ≥ 0. We can conclude from this brief game analysis that we cannot achieve our goal of having the country always be truthful and the COVAX Facility to always trust within this game design and given the game assumptions.

The truthfulness property of the VCG auction can tackle the issue of deceptive reporting since the incentive-compatibility property of VCG auctions ‘guarantees’ that each player would not benefit from being dishonest in reporting their readiness for vaccine distribution and thus each country would have a strong incentive to report their readiness truthfully. This is investigated in the next section.

## 4. Repeated VCG auction with artificial payments

The disproportionate distribution of COVID-19 vaccines has arguably stemmed from the selfish behaviour of high-income countries and from the lack of global governance. This research aims to design a global market that allocates vaccines fairly among countries regardless of their ability to pay by aligning the objectives of selfish countries with global social objectives. We propose a repeated auction mechanism with an artificial payment system to guarantee equitable access to vaccines and to provide monetary incentives for both high-income and low-income countries to report their true demand. Given that the main objective of vaccination is to protect populations against death, we assume that the main desirable objective of the proposed market is to minimise the number of deaths globally. This can be achieved by choosing an allocation mechanism that maximises the social welfare of all participating countries (c.f. Eq 9). Solving the social welfare optimisation problem can be done by eliciting the private utility information from all participating countries while ensuring that they report these private information truthfully. Optimal allocations and truthful reporting can be ensured by adopting the allocation and payment rules of *Vickrey-Clarke-Groves* (VCG) auction [45] for each round. In a VCG auction, participating agents are requested to report (bid) their private utilities, following which allocations are determined by optimising the social welfare (Eq 9). The payment rule in a VCG auction ensures that agents do not gain from untruthful bidding. This is called the incentive compatibility constraint:

$$\begin{array}{c} u_{i}\left({\hat{q}}_{i},{\hat{\theta }}_{i}\right)-{\hat{p}}_{i}\geq u_{i}\left(q_{i},\theta_{i}\right)-p_{i}\;\;\;\forall i\in N, \end{array}$$ (12)

where ${\hat{q}}_{i}$ and ${\hat{p}}_{i}$ are respectively the allocation and payment of agent *i* when they report their true type ${\hat{\theta }}_{i}$, while *q*_*i*_ and *p*_*i*_ are their allocation and payment when they report *θ*_*i*_ untruthfully. This means that the utility *u*_*i*_(*q*_*i*_, *θ*_*i*_) agent *i* gets from reporting their true type *θ*_*i*_ is never less than what they get from untruthful bidding *u*_*i*_(*q*_*i*_, *θ*). VCG uses the Clarke tax payment rule to ensure incentive compatibility. This rule states that each agent *i* should pay the externality they impose on all the other participating agents N\{i} (hereafter N-i), i.e. the loss in the welfare of all the other agents that is caused by the presence of agent *i*.

$$\begin{array}{c} p_{i}=\underset{q_{j}}{\text{max}}\;\sum_{j\in N-i}u_{j}\left(q_{j},\theta_{j}\right)-\sum_{\begin{array}{c} j\in N\\ j\neq i \end{array}}u_{j}\left(q_{j},\theta_{j}\right). \end{array}$$ (13)

The first term of this payment rule refers to the optimal welfare of agents N-i when agent *i* does not partake in the auction while the second term is the welfare of agents N-i when agent *i* participates. This payment rule aligns selfish behaviour with group-optimal behaviour by ensuring that agents report their private types truthfully to achieve their best self-interest. The proof is as follows. Assuming that selfish agents aim at maximising their benefit:

$$\begin{array}{c} \underset{\theta_{i}}{\text{max}}\;\left(u_{i}\left(q_{i},\theta_{i}\right)-p_{i}\right)=\\ \underset{\theta_{i}}{\text{max}}\;(u_{i}\left(q_{i},\theta_{i}\right)-(\underset{q_{j}}{\text{max}}\;\sum_{j\in N-i}u_{j}\left(q_{j},\theta_{j}\right)-\sum_{\begin{array}{c} j\in N\\ j\neq i \end{array}}u_{j}\left(q_{j},\theta_{j}\right))). \end{array}$$ (14)

Combining the first and third terms of this objective yields the social welfare. Thus the objective of the selfish agent becomes:

$$\begin{array}{c} \underset{\theta_{i}}{\text{max}}\;(\sum_{i\in N}u_{i}\left(q_{i},\theta_{i}\right)-\underset{q_{j}}{\text{max}}\;\sum_{j\in N-i}u_{j}\left(q_{j},\theta_{j}\right)). \end{array}$$ (15)

Given that the second term of this objective is independent of agent *i*’s report, it can be eliminated from this maximisation problem. Thus, the best strategy agent *i* can take to maximise their benefit is to ensure social welfare is maximised by reporting their type ${\hat{\theta }}_{i}$ truthfully.

We propose using the VCG auction as a fair allocation mechanism for distributing vaccines globally. This is due to its efficiency (optimal social welfare) and truthfulness (incentive compatibility). Nonetheless, it cannot be directly applied to the problem of vaccine distribution because it uses monetary payments to incentivise truthful bidding. This contradicts our aim to fairly distribute vaccines to countries regardless of their wealth. Inspired by [24], we take advantage of the periodical nature of the vaccine distribution problem and propose a repeated VCG auction with an artificial payment system that incorporates budget constraints.

**Fig 1:**
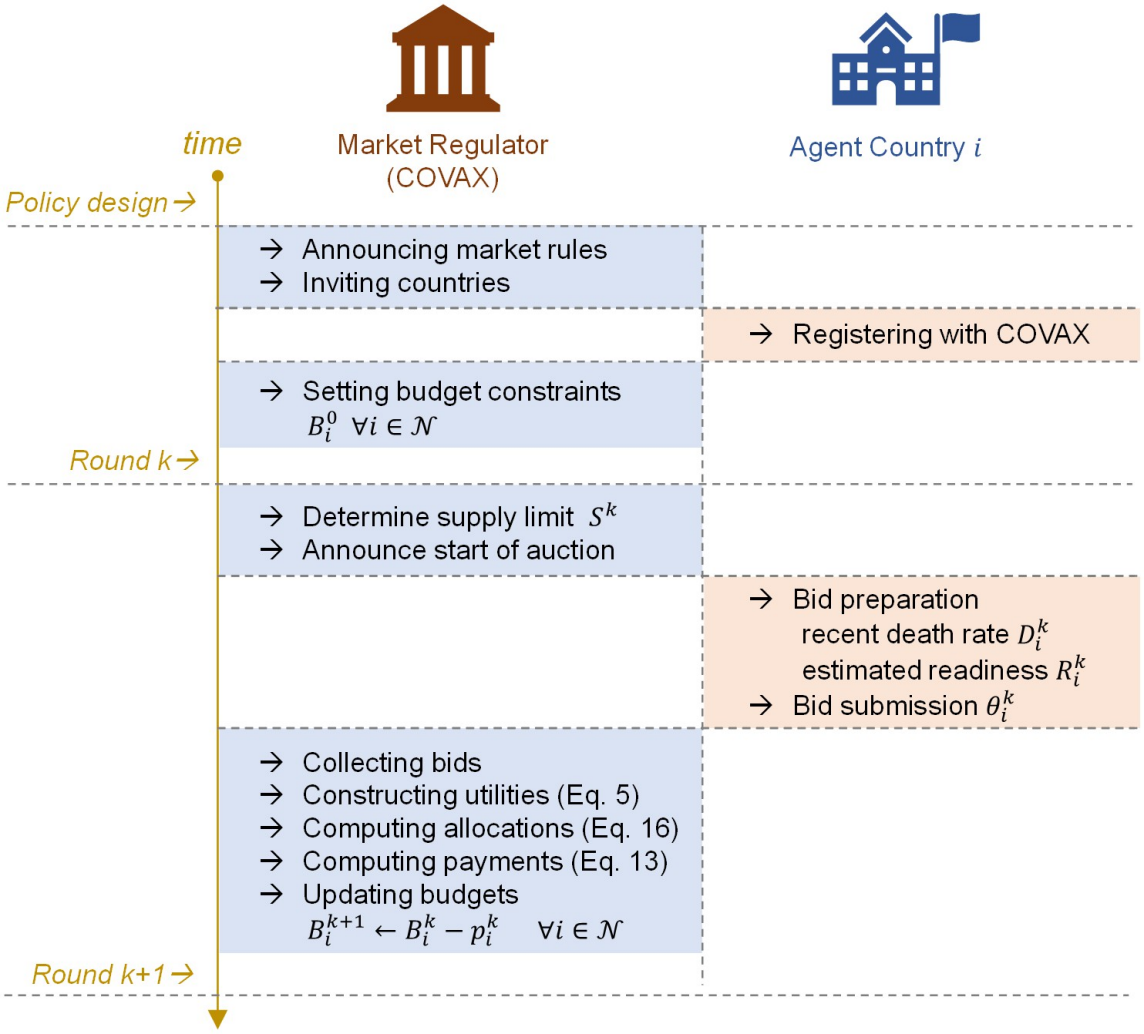
Flow of the proposed repeated auction, showing the sequence of operation and the interactions between COVAX and a participating country *i*.

Fig 1 shows the flow of the proposed auction in the context of COVAX and COVID-19 vaccine distribution. At the onset of an epidemic, a global market regulator should be established to govern the distribution and delivery of vaccines. The regulator is tasked with designing the market rules and inviting countries for registration. Once clinical trials of a viable vaccine begin and production capacities can be estimated, the regulator announces the number of rounds *K* and the duration of each round (e.g. 12 monthly auction rounds in a year). They then set the initial budget for the next set of rounds for each of the participating countries $B_{i}^{0}\;\;\forall i\in N$ (budget allocation methods are discussed in 7). At round *k*, the regulator determines its supply capabilities *S*^*k*^ for the duration of the upcoming round and asks participants to report their recent death rate $D_{i}^{k}$ and their estimated readiness $R_{i}^{k}$. It then uses the agents’ bids to construct their utility functions as per Eq 5 and computes the vaccine allocations that maximise social welfare:

$$\begin{array}{cc} \underset{q_{i}^{k}}{\text{max}} & \sum_{i\in N}u_{i}\left(q_{i}^{k},\theta_{i}^{k}\right), \end{array}$$ (16a)

$$\begin{array}{cc} \text{subject}\;\text{to} & \sum_{i\in N}q_{i}^{k}\leq S^{k}. \end{array}$$ (16b)

The payments for each round $p_{i}^{k}\;\;\forall i\in N$ are then calculated using Eq 13 and budgets for the remaining rounds are updated accordingly $B_{i}^{k+1}=B_{i}^{k}-p_{i}^{k}$. This concludes an iteration of the repeated auction.

Given that infection spread of an epidemic is a stochastic process [46], countries cannot manipulate their reports and risk losing a portion of their budgets in fear of needing them for subsequent rounds, especially that countries gain minimal utility when they receive more vaccination doses than what they are ready to administer. Thus, incentive compatibility still holds in the proposed allocation mechanism without payments.

## 5. Results

In this paper, we propose a fair global market for the distribution of vaccines. Motivated by the reported failures of COVID-19 vaccine distribution, we consider COVAX as the global market regulator and apply our allocation mechanism to COVID-19 vaccines. In order to have a consistent comparison between our mechanism and that of COVAX, we use COVAX allocations of Round 7 (September 2021) [25] as our reference. The reason behind selecting this round is that most of its participants were found to rely solely on COVAX for securing their vaccines [25].

### 5.1 Experimental setup

Since COVAX allocation decisions are made on a monthly basis, it is safe to assume that an iteration of the proposed auction has the duration of a month. Therefore, we use COVID-19 death data as reported in August 2021 [47] to calculate the recent death rates, where *D*_*i*_ is the total deaths reported in country *i* during August 2021 per one million of population. We use logistics performance index LPI data [48] to represent a country’s ability to distribute its allocated vaccines, while physicians per 1,000 people data [49] is used to indicate its ability to administer those vaccines. We combine both these scores to estimate how many vaccine doses countries can administer in a month, where we assume that a country with an aggregate score of two is ready to vaccinate 5% of its population. This was found consistent with vaccination coverage data reported in Germany and in the UK. Moreover, we assume that *α* and *β* of Eq 5 are 0.02 and 0.2 respectively. Using these, we are able to construct the utility function for all participating countries.

Four of the 46 countries that participated in Round 7 reported a maximum amount of doses they are willing to receive in this round. This was incorporated in our experiment as allocation constraints. The data and code used in this experiment can be accessed at [50].

### 5.2 VCG versus COVAX

**Fig 2:**
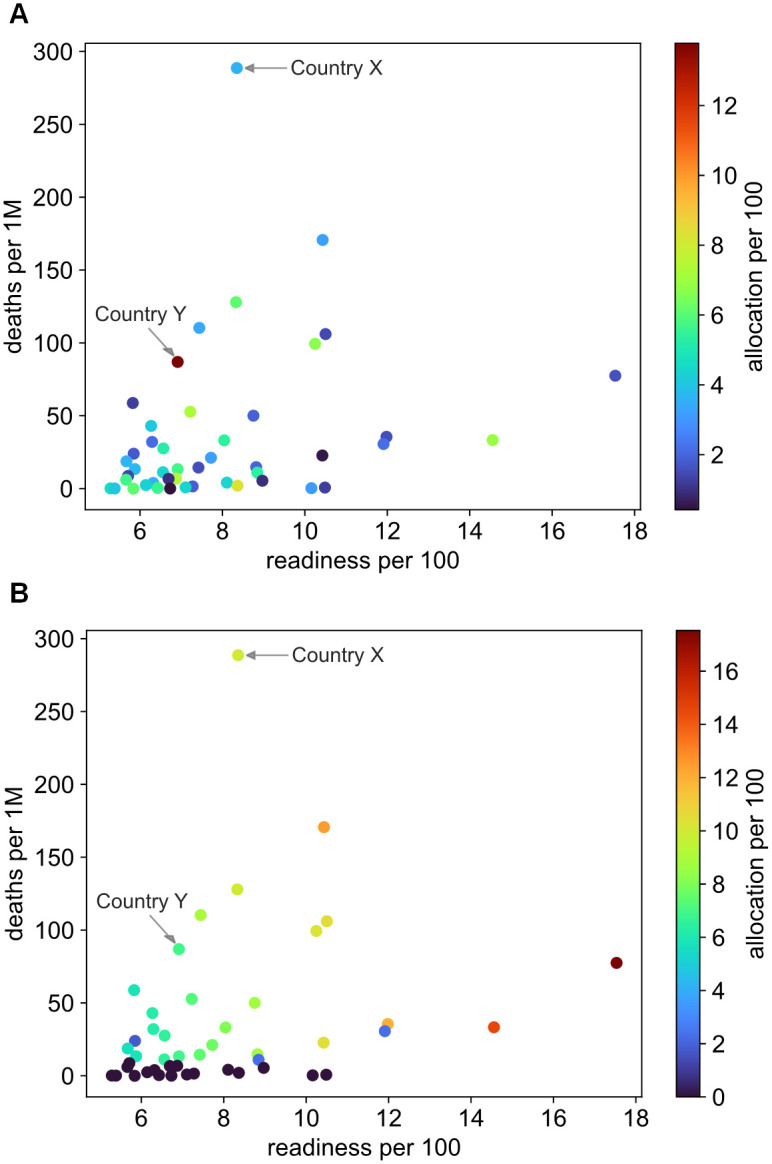
Comparison between the allocation decisions of COVAX and our proposed VCG mechanism for the countries participating in Round 7. (**A**) Covax allocations. (**B**) VCG allocations. Each dot represents a country drawn as per its recent deaths per 1M of population (vertical axis) and its readiness per 100 of population (horizontal axis), whereas the colour of each dot represents the allocation a country gets per 100 of its population.

The main objective of our proposed allocation mechanism is to reduce the number of deaths globally. We aim to achieve this by formulating the utility of receiving vaccines as a function of recent deaths and then maximising the sum of utilities. Wasted vaccines are also minimised by allowing minimal utility gains to those who receive more vaccines than their readiness. Fig 2A shows the allocation decisions made by COVAX in Round 7, while Fig 2B shows the allocations computed through the proposed VCG mechanism. These figures show the allocation a country gets as a function of both its recent death rate and its readiness. It is evident that the allocations made by COVAX are independent of the recent death rates, whereas the allocations computed using the proposed auction (hereinafter VCG allocations) are highly correlated with recent deaths. Indeed, when the VCG auction is applied in Round 7, any country with more than 11 deaths per 1M gets allocated however much it can administer in the next month.

In both scenarios, a few countries were given more doses than their readiness. In the COVAX Round 7 allocations, five countries were allocated an aggregate surplus of almost 1.5M doses, whereas in the VCG auction, four countries were given a surplus of 1.4M doses. Although the difference between both cases in surplus vaccines is only roughly 100k doses, the allocations made by VCG, including this surplus, are much more efficient than the ones made by COVAX. To demonstrate this, we will investigate the cases of country X and country Y shown in Fig 2. Country X has the highest death rate of this auction round with more than 280 deaths per 1M. Our proposed auction gives country X an allocation that is (1 + *β*) times its readiness, where *β* is the maximum vaccine surplus that can provide utility to the recipient countries (see Eq 5). This is believed to save the most lives as the most stricken countries can seek international aid to distribute and administer vaccines locally. In contrast, COVAX allocated Country X only 43% of its readiness. Awarding this difference in allocation to a country with a less death rate would lower the potential of the available vaccines in averting death. Country Y on the other hand, despite having a much lower death rate, received an allocation twice its readiness from COVAX. Since this may result in unnecessary storage costs at best, or in wasting those surplus vaccines at worst, it will also inhibit the capacity of those vaccines to avert deaths if allocated elsewhere.

**Fig 3:**
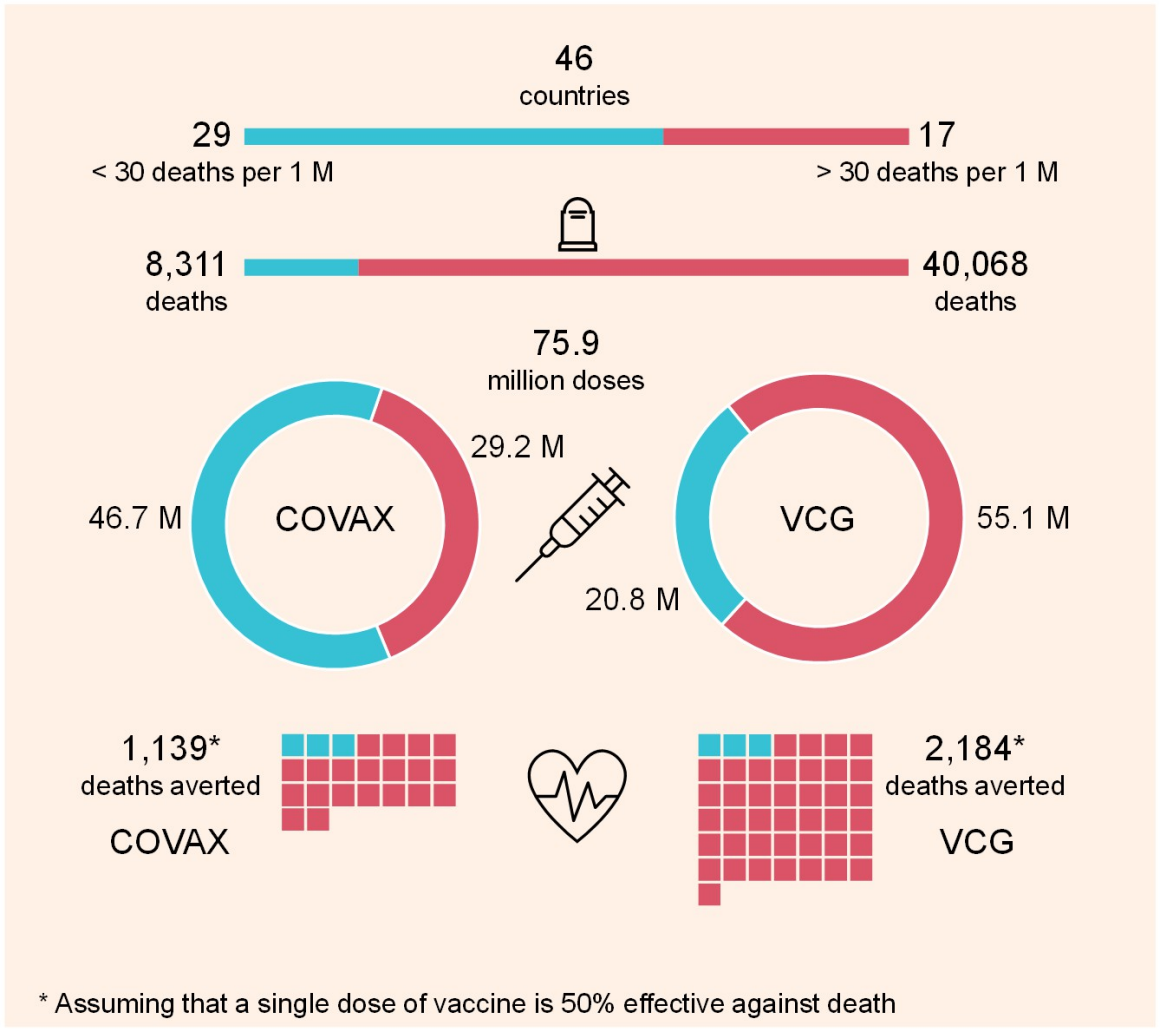
Deaths averted Covax vs. VCG. The countries that reported less than 30 deaths per 1M are denoted in blue while those that reported more than 30 deaths per 1M are denoted in red.

To evaluate the efficiency of the proposed market in averting deaths, Fig 3 demonstrates its performance in reference to that of COVAX for the allocations of Round 7. 46 countries took part in COVAX Round 7. Of those, 17 countries, denoted in red, had reported more than 30 deaths per 1M in the month prior to that of the COVAX decision announcement. A total of 40,068 deaths were reported in those countries, while 8,311 deaths were reported in the remaining 29 countries, denoted in blue. In the VCG auction mechanism, those 17 countries were allocated 73% of the available 75.9 M doses, whereas their share of those vaccinations was only 38% from COVAX. This illustrates the wasted opportunity of COVAX allocations.

Assuming that the death rate remains constant over the period of the round duration, and that a single dose of vaccination is 50% effective against death in expectation regardless of the producer, the proposed allocation mechanism results in 2,184 deaths averted, almost double what the COVAX allocation decisions achieve. This shows that if an efficient mechanism such as the proposed repeated auction is adopted by COVAX, then the objective of vaccine distribution, i.e. minimising deaths, can be achieved. As the vaccine effectiveness against death increases, the number of averted deaths grows faster in the case of VCG wen compared with the COVAX allocations. VCG will avert 437 deaths for each 10% increase in vaccine effectiveness (e.g., form 50% to 60%), while COVAX allocations would only avert 228 deaths. This is because the proposed VCG allocates vaccines efficiently to the countries most affected by the epidemic.

**Fig 4:**
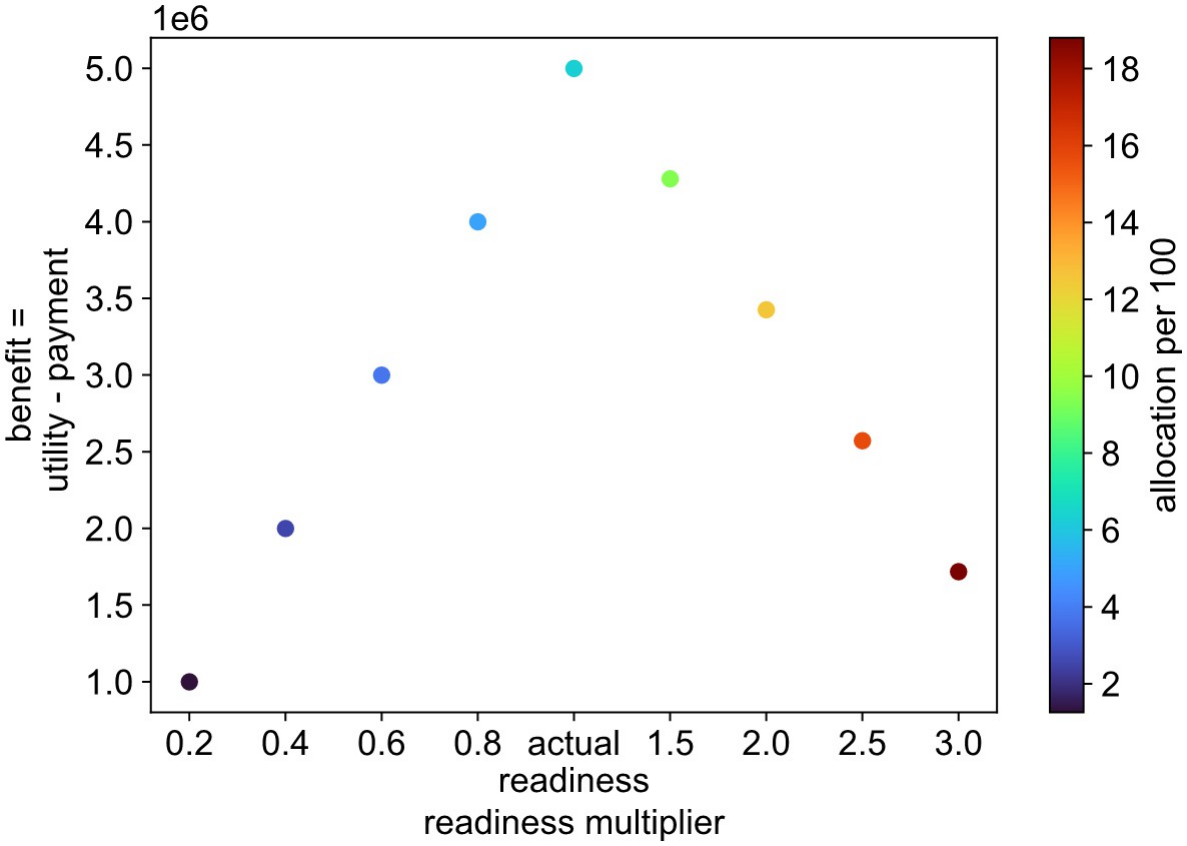
The benefit of receiving vaccines when a country misreports its readiness. The horizontal axis represents the multiplier by which readiness is altered while the vertical axis represents the difference between the utility of getting vaccines and the payment for these vaccines. The allocation a country gets by misreporting its readiness is normalised per 100 of population and is denoted by the colour of each data point.

Our proposed repeated auction adopts an artificial payment system, where each country is allocated a budget for a number of rounds, and at each round that budget is decremented by the VCG payment computed in that auction iteration. This method ensures that countries are incentivised to report their true type *θ* (i.e. recent deaths and readiness), which assert that efficient allocations can be made. In this experiment, we test this claim for a random country and plot in Fig 4 its benefit (i.e. difference between utility and payment) against a number of false readiness reports. Although this country can get higher allocations if it misreports its readiness, the most benefit it can achieve is when it reports the actual amount of vaccines it is ready to administer. i.e. the utility gain this country would get from this increase in allocation would be lower than the extra payment it would have to make. Thus reporting a higher readiness would result in a much decreased budget which in turn would affect its allocations in subsequent rounds.

**Fig 5:**
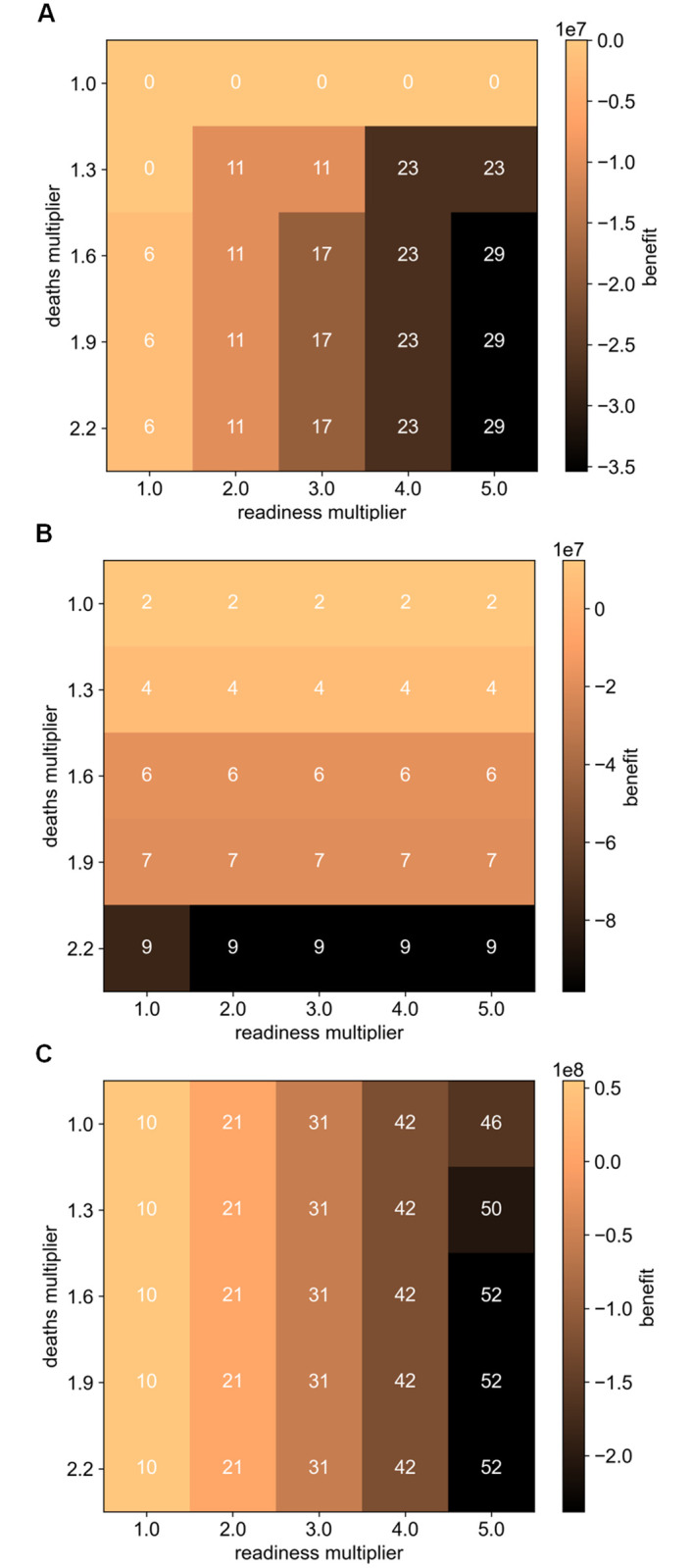
Effect of misreporting readiness and deaths on the benefit (difference between utility and payment) a country gets from receiving vaccines. Each cell represents a (*D*, *R*) report and its colour represents the benefit a country gets when submitting that report. Cell annotations represent the normalised allocations per 100 of population. (**A**) A country with low death rate. (**B**) A country with median death rate. (**C**) A country with high death rate.

Fig 5 demonstrates the incentive compatibility property of the proposed auction. Here, the benefit of a country is shown when it exaggerates both its recent deaths and its readiness. For each manipulated (*D*, *R*) report, the allocation and payment of an agent are computed through the proposed mechanism. The allocation for each (*D*, *R*) report, represented by a cell in Fig 5, are normalised per 100 of population and annotated on its cell. Cell colour represents the difference between the utility an agent gets from receiving vaccines and the payment it has to make for these vaccines. Fig 5A shows that a country with a low death rate would not receive any allocation unless it reports its deaths falsely. Nonetheless, the figure shows that it would be at loss if it does so (since the payment exceeds the utility of receiving the extra vaccines). Fig 5B shows that the effect of misreporting readiness in a country with a median death rate is negligible as it would not influence its allocation. Although exaggerating its deaths results in a higher allocation, it lowers its overall benefit. This effect is mirrored when a country has a relatively high death rate, shown in Fig 5C. Here, exaggerating deaths would not affect its allocation, but exaggerating readiness would, despite causing a loss in benefit. These figures demonstrate that countries, acting in their best self-interest, cannot achieve a better outcome by misreporting their private characteristics. Thus, proving that truthful reporting is a dominant strategy in the proposed repeated auction.

## 6. Discussion

Our proposed mechanism achieves utilitarian division that maximises social benefit fulfilling the fairness criterion we set out in Section 2. Additionally, we were able to incentivise accurate reporting of countries’ readiness and deaths by designing a mechanism that optimises the outcome of countries that do so, whilst penalising those that do not. We have also shown that our mechanism, based on the VCG auction, would result in a greater number of averted deaths than the mechanism used by COVAX.

The reliance of our mechanism on readiness and deaths in the determination of allocation raises several potential issues. First, countries that are most ‘ready’ would likely be wealthier countries, which may result in more vaccines being allocated based on countries’ wealth. Given that readiness is largely a function of a country’s storage capacity, infrastructure quality, and the condition of its healthcare system, it is crucial that efforts are made to improve readiness in all countries where that is needed. Second, declaring that a death was due to COVID-19 requires that the patient have obtained a recorded diagnosis before they die. This is more likely to occur in wealthier countries with more developed healthcare systems, which—again—may result in a mechanism that favours rich countries that are better able to record and report COVID-19 cases and deaths. However, these two considerations would need to be balanced against the greater benefit we have demonstrated that can be derived by adopting this mechanism. Thirdly, given that in some countries, a large number of deaths go unidentified or unreported, this may mean that true demand may be underestimated for these countries in our model. It is therefore crucial that testing and reporting capacity is concurrently supported as part of the pandemic response.

Taking a broader view than the details of the distribution mechanism we have proposed, the potential implementation of *any* equitable vaccine distribution mechanism faces several major challenges. The transnational nature of pandemics means that the individual benefit derived by states from hoarding vaccines and pursuing excessive bilateral contracts with vaccine producers, will only prolong the pandemic. This sub-optimal outcome for the collective caused by the pursuit of individual interest is characteristic of collective action problems. Therefore, the establishment of robust institutions in advance of future pandemics will be key to guiding this process of international cooperation.

The literature on international organisations has identified factors that predict the success of cooperation. Firstly, the quality of the information that actors receive about their counterparts’ behaviour. Secondly, the frequency of interactions. Frequently iterated interactions are more likely to foster robust cooperation. Thirdly, the relative vulnerability of participants. If participants are all equally vulnerable to a problem, this should yield successful cooperation. Unequal vulnerability on the other hand yields lower incentives for cooperation amongst those not as vulnerable. Fourthly, a higher number of actors make cooperation more challenging. Finally, the scope of an issue: i.e., whether it is a single-issue or multi-faceted. The more multi-faceted an issue is, the lower the chance of successful cooperation [51]. Pandemic management performs poorly on all these parameters; it involves a situation with many state and sub-state actors interacting intermittently (due to the rarity of pandemics), with imperfect information about their counterparts and many different considerations to make beyond public health (economic, social, etc.). Furthermore, the vulnerability of states to pandemics varies massively due to their geography, demographics, and many other factors.

Ultimately, the success or failure of a distribution mechanism depends on the willingness of states and sub-state actors to engage in cooperative activity or the ability of international institutions to compel them to do so. A major step that can reduce the complexity of distributive decisions is increasing global vaccine supply through simplifying and expediting the process of obtaining waivers on sections of the World Trade Organisation’s (WTO) agreement on Trade-Related Aspect of Intellectual Property Rights (TRIPS) can help to achieve this. This will allow manufacturers to produce a vaccine without risking legal ramifications, thus increasing global supply, and ensuring a greater geographic distribution of manufacturing. Measures to weaken intellectual property can disincentivise investment by pharmaceutical companies in vaccines. However, this measure is not without precedent; HIV medications are currently produced in multiple countries on a TRIPS waiver.

## 7. Conclusion

In this paper, we used game theory to gain insight into the problem of global vaccine distribution. We proposed a fair market mechanism that can align ‘selfish’ objectives with optimal global objectives. The interaction between a central global vaccine provider (e.g. COVAX facility) and a country reporting its demand was modelled as a two-player game. The analysis of the Nash and mixed Nash equilibria of that game has shown that countries, seeking their best self-interest, might exaggerate their vaccine readiness. Thus, we have proposed a truthful repeated auction mechanism for the global distribution of vaccines. Each round of this repeated auction is based on VCG auction where countries’ allocations are determined that maximise the social benefit of administering vaccines. The proposed market mechanism employs an artificial payment system where budgets allocated at the market initialisation phase are decremented at each auction round. Compared with the allocation decisions made by COVAX in Round 7 (announced on 17 September 2021), our proposed allocation mechanism showed superior potential in avoiding deaths. Our results have also demonstrated that truthful reporting of deaths and readiness is a dominant strategy.

The uncertainty of infection spread in an epidemic coupled with the non-linearity of the VCG payment rule makes it difficult to specify a budget allocation method. Nonetheless, it is believed that any fair budget allocation method should satisfy the following rules: (i) Budgets are inversely proportional to the gross domestic product GDP per capita $B_{i}^{0}\propto 1/GDP_{i}$; and (ii) Budgets are directly proportional to population $B_{i}^{0}\propto population_{i}$.

These rules ensure that low-income countries with large populations are allocated sufficient budgets that would cover their vaccine requirements in a set of rounds. Whereas high-income countries would be allocated low budgets and then would have to pay in order to receive their allocated vaccines. We believe that a trial period would provide practical information that would help in refining the budget allocation method. Indeed, pilot implementation of this mechanism before any future pandemics would help prove its viability and identify potential issues. The current vaccination drive led by COVAX presents an opportunity to do this.

One of the limitations of the proposed vaccine distribution mechanism is that it does not account for the objectives of vaccination other than minimising deaths. Modelling the vaccine distribution problem as a multi-objective optimisation problem can be advantageous, especially for epidemics that affect hospitalisations more severely than deaths. Another limitation of our proposed market is the assumption that vaccination is a homogeneous product whereas different manufacturers produce vaccines with different characteristics, such as required number of shots and efficacy. Proposing an envy-free division of vaccines produced by different manufacturers as a heterogeneous good is a possible future research direction. Another possible future research direction is accounting for the production and transportation costs in the optimisation of global vaccine distribution. Future research can also focus on modelling the utility of vaccines more accurately, by combining the mortality rate with other measures such as years of life lost and lost quality-adjusted life years [52].
